# The Diagnostic Utility of Contrast-Enhanced FLAIR Imaging in the Diagnosis of Pediatric Uveitis

**DOI:** 10.5334/jbsr.3565

**Published:** 2024-06-28

**Authors:** Saffet Ozturk, Esin Kurtulus Ozturk, Yasemin Tasci Yildiz, Bahadir Konuskan

**Affiliations:** 1 Ankara Etlik City Hospital, Department of Radiology, Ankara, Turkey; 2 Ankara Etlik City Hospital, Department of Radiology, Ankara, Turkey; 3 Ankara Etlik City Hospital, Department of Pediatric Radiology, Ankara, Turkey; 4 Ankara Etlik City Hospital, Department of Pediatric Neurology, Ankara, Turkey

**Keywords:** Uveitis, childhood, magnetic resonance imaging, FLAIR

## Abstract

*Objectives:* Contrast-enhanced FLAIR fat-suppressed (CE-FLAIR-FS) imaging can potentially increase the diagnostic accuracy of uveal diseases and ultimately provide better patient management. This study aimed to determine the diagnostic value of CE-FLAIR-FS imaging versus contrast-enhanced T1-weighted imaging (CE-T1WI) in the assessment of pediatric patients with uveitis.

*Material and methods:* Twenty-one children with uveitis who underwent whole brain magnetic resonance imaging (MRI), including CE-FLAIR-FS and CE-T1WI, were retrospectively included in the study. We evaluated the presence of uveal tract contrast enhancement with thickening, vitreous humor signal abnormality, and accompanying brain abnormalities. The uveal enhancement intensity was assessed semiquantitatively as mild, moderate, and marked uveitis compared to CE-T1WI and CE-FLAIR-FS images.

*Results:* Panuveitis (61.9%) was the most frequent anatomic location, and most of them were idiopathic (47.6%). Of the 42 eyes with clinical uveitis, enhancement of the uveal tract was observed on CE-FLAIR-FS images in 21 eyes (50%), while in 5 eyes (11.9%) on CE-T1WI. The sensitivity of CE-FLAIR-FS in panuveitis was detected to be quite high (80.8%). The number of affected eyes and enhancement degree were found to be higher on CE-FLAIR-FS (*p* < 0.001). In assessing the severity of uveitis, CE-FLAIR-FS grades were significantly higher and more sensitive than CE-T1WI (*p* < 0.001, *Z*: −4.347). Three patients had vitreous abnormal signals on CE-FLAIR-FS images, but none on CE-T1WI.

*Conclusion:* CE-FLAIR-FS plays a significant role in the diagnosis of pediatric uveitis, identifying the involvement and severity of the uveal inflammation and guiding the appropriate management. It would be beneficial to add it as a standard sequence to the routine MRI protocol for uveal pathologies.

## Introduction

Uveitis is a sight-threatening intraocular inflammation that can damage at least one layer of the eye, the retina or vitreous, in conjunction with the adjacent uveal tract [1]. Uveitis is a relatively uncommon condition in children compared to adults. Anterior uveitis is the most common type in children, is often asymptomatic, and tends to become chronic, leading to ocular damage more frequently [2, 3].

Pediatric uveitis, which tends to be chronic and recurrent, may not heal fully between attacks and can cause many irreversible complications. Therefore, most authors believe that children are more prone to complications than adults. Since it is often resistant to treatment, it increases the risk of developing vision-threatening complications such as cataract, glaucoma, amblyopia, band keratopathy, and sequelae in visual function [3, 4].

Magnetic resonance imaging (MRI) is a valuable imaging technique that objectively reveals the features of uveitis and accompanying neurological abnormalities [5,6]. Recent studies have reported that contrast-enhanced fluid-attenuated inversion recovery (CE-FLAIR) sequences can demonstrate even low concentrations of gadolinium uptake by suppressing leptomeningeal and brain parenchymal abnormalities with fluid along with vascular traces [7, 8]. Contrast-enhanced FLAIR fat-suppressed (CE-FLAIR-FS) images can better reveal the abnormal pathological uveal contrast enhancement compared to contrast-enhanced T1-weighted imaging (CE-T1WI).

To our knowledge, this is the first study in the English literature describing the diagnostic value of CE-FLAIR-FS imaging in pediatric uveitis. Herein, we aimed to investigate the feasibility and clinical utility of CE-FLAIR-FS imaging compared to the widely used CE-T1WI in pediatric patients with active uveitis.

## Material and Methods

### Study design and patient selection

Between January 2017 and September 2022, we retrospectively reviewed the medical records of uveitis cases. The patients had complaints of headache and blurred vision, and a brain MRI was performed to reveal the neurological diseases accompanying acute uveitis. All the cases underwent cranial MRI, and the radiologic images were retrieved from the institution’s picture archiving and communication system (PACS). The study population was identified according to the following inclusion criteria: (a) patients under the age of 18 years, (b) patients with active uveitis (acute uveitis, first or recurrent episodes), and (c) patients with available contrast-enhanced MRI. Individuals with a history of orbital surgery, ocular malignancy, or contraindications for MRI and contrast were excluded from participation. Seventy-one pediatric patients were referred with a clinical diagnosis of uveitis. We assessed all patients for eligibility, and a total of 50 cases were excluded from the study due to the following reasons: 37 patients were excluded for the absence of contrast agent use, 10 patients for the absence of CE-FLAIR-FS sequences, and 3 patients for MRI motion artifacts. On the other hand, 21 patients with a diagnosis of uveitis met the inclusion criteria and were included in the study.

### MRI protocol and analysis

MRI scans were obtained less than 1 week from the onset of each uveitis symptom. All contrast-enhanced cranial MRIs were performed on a 1.5 Tesla MRI scanner (GE Healthcare, Milwaukee, WI, USA). A series of routine MRI sequences were performed, including axial and sagittal T1-weighted imaging (T1WI) and fluid-attenuated inversion recovery (FLAIR), as well as axial and coronal T2-weighted imaging (T2WI), and diffusion-weighted imaging (DWI). Additionally, post-contrast (Gadolinium-DTPA) axial and sagittal T1WI and axial FLAIR-FS images were performed. The CE-T1WI imaging (TR: 500, TE: 9, FOV: 220 mm, slice thickness: 5 mm, slice interval: 1.5 mm, and acquisition time: 3 min) and the CE-FLAIR-FS imaging (TR: 9000, TE: 93, TI: 2500, FOV: 220 mm, slice thickness: 5 mm, slice interval: 1.5 mm, and acquisition time: 4 min) commenced at 2 minutes 30 seconds, and about 8 minutes after intravenous gadolinium contrast administration, respectively.

**Figure 1:**
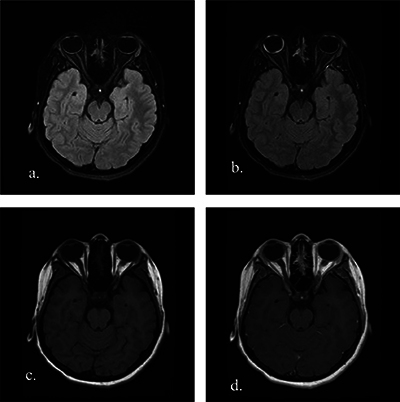
Bilateral panuveitis in a 13-year-old girl with Behcet’s disease. Axial fat-suppressed FLAIR non-enhanced **(a)** and contrast-enhanced **(b)** images reveal uveal tract thickening with marked contrast enhancement in the right eye and mild contrast enhancement in the left eye. The increased vitreous signal in the right eye is also noted. Axial non-enhanced **(c)** and contrast-enhanced **(d)** T1WI show no definite uveal tract enhancement in both eyes.

All images were analyzed retrospectively in consensus by two experienced radiologists (experienced 7 and 9 years, respectively). We documented the presence of uveal tract thickening, uveal tract contrast enhancement, vitreous humor signal abnormality, and diffusion properties. Uveal tract enhancement was considered positive when the uveal signal intensity was higher than adjacent extraocular muscles (Figure 1).

**Figure 2:**
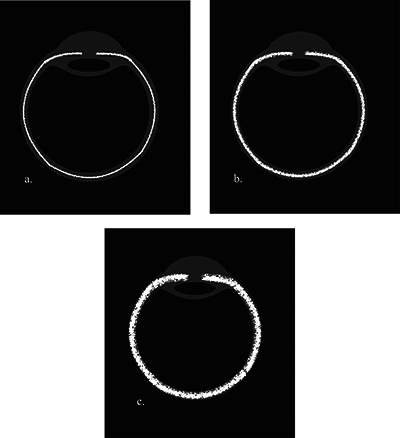
Schematic drawing of the semi-quantitative analysis of uveitis severity: mild **(a)**, moderate **(b)**, and marked **(c)** uveal tract enhancement.

**Figure 3:**
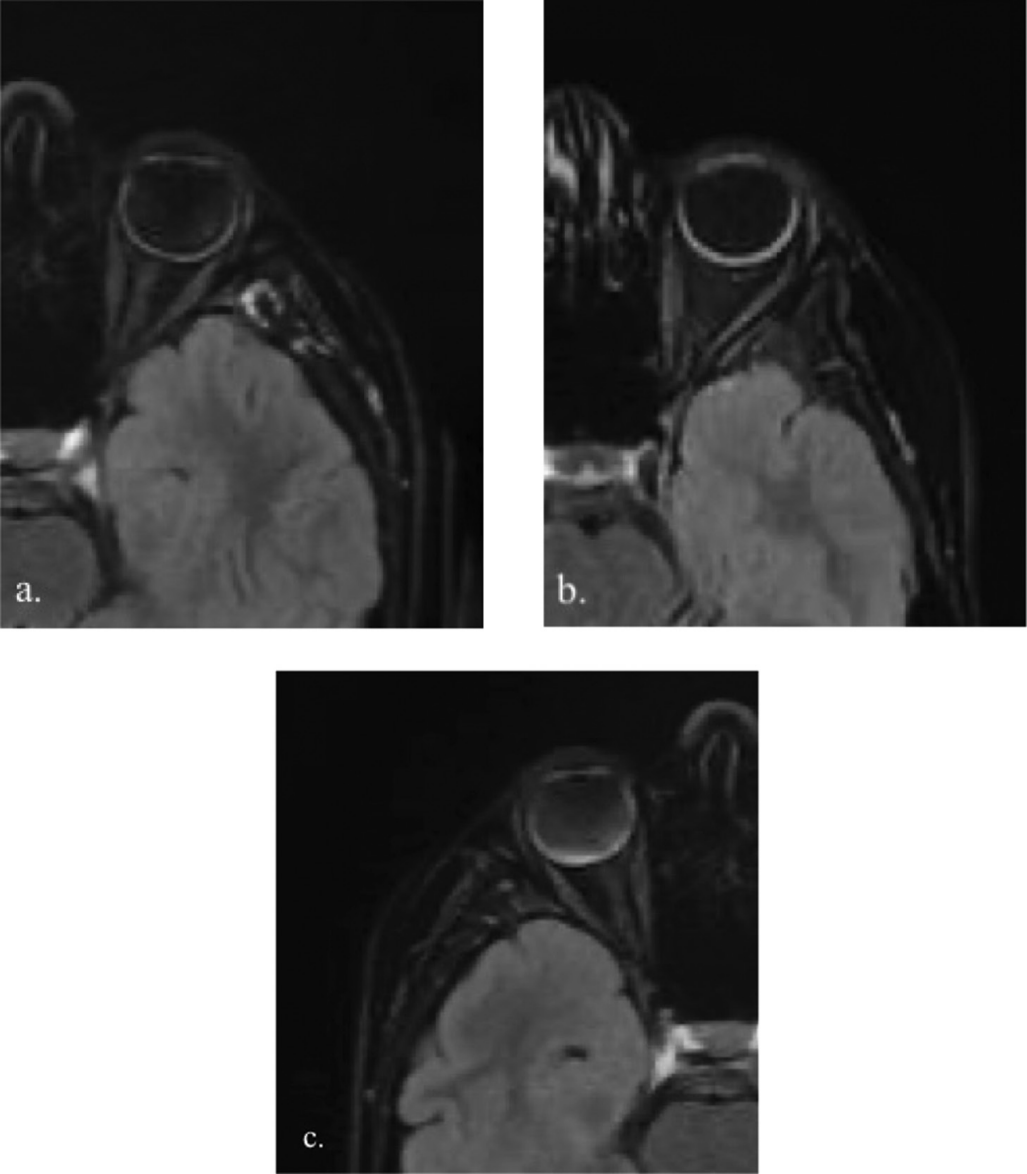
Semi-quantitative assessment of uveitis using CE-FLAIR-FS imaging: mild **(a)**, moderate **(b)**, and marked **(c)** uveal tract enhancement.

The site and location of contrast enhancement (anterior, posterior, panuveitis, and pars planitis) and enhancement intensity were also noted. The degree of uveal tract enhancement was categorized semiquantitatively as mild, moderate, and marked on CE-T1WI and CE-FLAIR FS images (Figures 2 and 3).

### Statistical analysis

Statistical data analyses were performed using the IBM SPSS Statistics software, version 24.0 (SPSS Inc., Chicago, IL, USA). All continuous variables were described using the median with an interquartile range for nonparametric data and the mean, and standard deviation for parametric data using the Kolmogorov–Smirnov test.

The normality of the distribution of the data was verified with the Shapiro–Wilk test.

Among total patients and patients with panuveitis, the sensitivity of the CE-T1WI and CE-FLAIR-FS images was calculated by ophthalmologic slit lamp exams as a reference standard. A *p* < 0.05 was considered significant. The presence and grading of uveal tract contrast enhancement were compared using Chi-square and the Wilcoxon signed-rank test.

This retrospective study was approved by the institutional review board of our institution (E-22/09-438). Written informed consent was obtained from all participants and their parents.

## Results

In this study, out of 71 patients with a clinical diagnosis of uveitis, 21 patients (42 eyes) had adequate MRI workup, including CE-T1WI and CE-FLAIR-FS sequences. The age of the patients included in the study ranged from 9 to 17 years (mean age 12.7 ± 2.26 years) at the time of uveitis diagnosis.

The patients were 15 (71.4%) females and 6 (28.6%) males. Panuveitis was the most common anatomic location in 13 (61.9%) patients, followed by intermediate uveitis in 5 (23.8%) patients, and anterior uveitis in 3 (14.3%) patients.

The most frequent disease found was Behcet’s disease with six patients (28.6%), followed by juvenile idiopathic arthritis (JIA) with five patients (23.8%). The remaining 10 cases (47.6%) were idiopathic uveitis because their etiologies could not be determined (Table 1).

**Table 1:** Patients clinical characteristics.

	*N (%)*
**Number of patients**	21
**Age-year, mean ± (range)**	12.7 ± 2.26 (9–17)
**Gender** Female Male	15 (71.4%) 6 (28.6%)
**Anatomic location of the uveitis** Anterior Posterior Intermediate/pars planitis Panuveitis	3 (14.3%) 0 5 (23.8%) 13 (61.9%)
**Etiology of uveitis** Idiopathic Behcet’s disease Juvenile idiopathic arthritis	10 (47.6%) 6 (28.6) 5 (23.8%)

We evaluated the MRI of 21 uveitis patients with 42 eyes. On CE-FLAIR-FS images, 21 eyes (50%) showed uveal tract enhancement, while uveal enhancement was found in only 5 eyes on CE-T1WI. Of the patients, seven had bilateral uveal enhancement, and six had monocular enhancement (four right-sided, two left-sided) on CE-FLAIR-FS images. In all patients with anterior or intermediate uveitis and one case with panuveitis, no pathological enhancement was observed in both post-contrast images. All patients with enhancement patterns were presented with panuveal contrast enhancement. No restriction diffusion was detected in globes and ocular fat planes. Three patients had a vitreous abnormal signal on CE-FLAIR-FS images, however, no patients on CE-T1WI. In eyes with uveitis, CE-FLAIR-FS sensitivity was 50%, while in eyes with panuveitis, it was detected at a considerably higher rate (80.8%). On CE-T1WIs, sensitivity was significantly lower in both total and panuveitis (11.9% and 19.2%, respectively) (Table 2).

**Table 2:** Sensitivity of CE-FLAIR-FS and CE-T1WI.

	SENSITIVITY	
	TOTAL UVEITIS	PANUVEITIS
Postcontrast T1WI	11.9%	19.2%
Postcontrast FLAIR-FS	50 %	80.8%

Compared to the evaluation with CE-T1WI, the number of affected eyes and enhancement degree were found to be higher with CE-FLAIR-FS images (*p* < 0.001).

For the comparison of the grading of the uveal tract enhancement, CE-FLAIR-FS grades were significantly higher and more sensitive than CE-T1WI (*p* < 0.001, *Z*: −4.347) (Table 3).

**Table 3:** Comparison of presence and degree of uveal tract contrast enhancement.

	*CE-FLAIR-FS N (%)*	*CE-T1WI N (%)*	P VALUE*
**Total eye** **Uveal tract contrast enhancement** **Presence of vitreous abnormalities**	42 21 (50%) 3 (7.1 %)	42 5 (11.9%) 0	**<0.001** –
**Enhancement degree** Mild (+) Moderate (++) Marked (+++)	15 4 2	5 0 0	<0.001**

* *P < 0.05.*

** *Wilcoxon signed the rank test, Z.*

There was no abnormality in the optic nerves, subretinal, or subchoroidal areas. Additionally, no evidence of cerebral lesions, including demyelinating white matter lesions, neurodegenerative disease was identified.

## Discussion

Uveitis is relatively uncommon in the pediatric population, accounting for 5% to 10% of all uveitis cases. A greater proportion of girls than boys are affected by this condition. Pediatric uveitis is relatively rare, accounting for 5% to 10% of all uveitis cases, and affects girls more frequently than boys [2]. Children with uveitis are often asymptomatic or may not be able to express their possible complaints, which may cause a delay in timely diagnosis and treatment. Delayed diagnosis may lead to worsening clinical outcomes and increased severity of the disease [9, 10]. When compared to the findings previously reported in the literature, it was observed that similarly, our cases were mostly girls and were idiopathic. Approximately 8% of patients with uveitis may develop neurological symptoms during the course of the disease. Neuroimaging is often needed to investigate possible accompanying brain pathologies in children with uveitis and neurological symptoms [5]. The patients included in the present study had undergone a brain MRI due to the presence of neurological symptoms. Most of our cases presented with panuveitis and headache. No abnormal findings were found in their brain parenchyma. We thought that neurological symptoms may occur more frequently in patients with panuveitis.

The prevalence of etiology and types of uveitis vary depending on ethnic and geographical distribution [2, 3]. In a recent multicenter study from Turkey, the most common systemic association in the Turkish population was reported to be JIA in younger onset and Behçet’s disease in older children [11]. In our study, all of our cases had noninfectious uveitis. It was mostly over 10 years old and mostly associated with Behcet’s disease. A correlation was identified between anterior uveitis and JIA in all patients.

The diagnosis of uveitis is predominantly based on clinical diagnosis; it involves a comprehensive history, a physical examination, laboratory blood analysis, and slit-lamp biomicroscopy. The literature on MRI findings of uveitis is quite limited, consisting of a small number of cases and case series. MRI findings of uveitis have been reported as increased uveal enhancement and/or abnormal uveal tract thickening, subretinal effusions, and vitreous humor signal abnormalities, with no DWI restriction [11, 12].

On imaging, inflammatory or infectious scleritis appears similar to uveitis, regardless of the underlying disease, and is difficult to distinguish from uveitis. The presence of periscleral cellulitis and scleral thickening—enhancement favor the diagnosis of scleritis. Moreover, nodular uveitis needs to be differentiated from tumors. Clinical inflammatory signs and a lack of diffusion restriction on MRI should indicate uveitis [12].

Li et al. described the MRI and CT findings of seven adult uveitis cases and reported that uveal tract enhancement was a common finding in patients with uveitis, regardless of anatomic distribution and etiology. However, in this case series, the researchers only evaluated the patients with postcontrast T1WI [6].

Herrera et al. conducted the first and only study in the literature comparing CE-FLAIR-FS versus CE-T1WI in five adult uveitis cases and studied at both 1.5 and 3T. They concluded that CE-FLAIR-FS imaging was more sensitive than CE-T1WI in the evaluation of uveitis [13]. Our study is the first report of the utility and accuracy of CE-FLAIR-FS imaging in the diagnosis of pediatric uveitis. Similar to the findings of Herera et al., CE-FLAIR-FS imaging was superior to contrast-enhanced T1 imaging for detecting uveal tract enhancement.

The sensitivity of CE-FLAIR-FS sequences was 50% in all pediatric uveitis cases and 80.8% in panuveitis cases, while the sensitivity of CE-T1WI sequences was lower in both all cases and panuveitis cases, 11.9% and 19.2%, respectively. In all anterior uveitis, we found that ocular enhancement was not detected in both the CE-T1WI and CE-FLAIR-FS sequences. Vitreous signal abnormalities in three (7.1%) patients were observed in only CE-FLAIR-FS imaging. Recent studies have reported that younger age onset and uveitis involving the posterior segment have a higher risk of complications and are associated with poor prognosis [14, 15]. Therefore, early diagnosis and treatment of posterior uveitis and panuveitis are important to prevent complications that may lead to vision loss. In our study, we found that the sensitivity of CE-FLAIR-FS imaging was significantly higher, especially in panuveitis.

Herrera et al. also stated that contrast enhancement in the uvea on 3T imaging in 10 healthy control patients could be a normal finding with a prevalence of 90% [13]. Whereas the uveal tract enhancement has been reported at a rate of 3% to 6% in the normal population in CE-FLAIR-FS imaging at 1.5T [13, 16]. All our images were obtained using the same protocols on a 1.5T MRI device. Thus, we achieved homogenization in the imaging and technical evaluation of the patients. Therefore, the radiologist must be careful and knowledgeable about this issue before reporting any uveal enhancement as pathological. In our study, contrast enhancement accompanied by uveal thickening was evaluated as pathological uveitis in order to prevent false positivity.

In their study, Li et al. reported a single case of adult posterior uveitis exhibiting restriction within the uvea and effusion on DWI [6]. In our cases, no ocular diffusion restriction or accompanying brain abnormalities were detected. Furthermore, we demonstrated that semiquantitative grading of uveal contrast enhancement can be employed to ascertain the severity of the disease. We found that the degree of contrast enhancement in CE-FLAIR-FS was statistically significantly higher than in postcontrast T1 images (*p* < 0.001).

A high degree of uveal enhancement may be considered a high risk of complications, and semiquantitative grading may play an important role in disease management.

It is important to note that the study is limited by its retrospective single-center design, its relatively small sample size, and the variability inherent in the patient population. The heterogeneity of uveitis etiology might have affected the results of uveal contrast enhancement since the anatomic affected location and severity of each specific disease. The lack of previous research studies represents a potential limitation to the accuracy of MRI findings in pediatric uveitis. Additionally, the results of this study may not be generalized to all children with uveitis, as the etiology may vary by race or ethnic origin. Uveitis diagnosis as study inclusion criteria was purely based on clinical symptoms, which may significantly influence the results since many uveitis are asymptomatic. One of the important limitations is the subjective and semi-quantitative assessment of the degree of uveal tract enhancement.

In conclusion, CE-FLAIR-FS imaging was observed with significantly greater sensitivity than conventional CE-T1WI for the detection of uveal tract enhancement in pediatric patients with uveitis. CE-FLAIR-FS imaging seems to be a promising diagnostic imaging method and a valuable adjunct to conventional MRI as a standard sequence for assessing uveal pathologies in pediatric uveitis. Semiquantitative analysis of uveal tract enhancement can be used to assess the severity of uveitis and predict disease progression and prognosis. A multicenter randomized controlled trial is required to assess the utility of the CE-FLAIR-FS sequence in the assessment of pediatric uveitis.
